# Remuneration of General Practitioners

**Published:** 1830-04-01

**Authors:** 


REMUNERATION OF GENERAL PRACTITIONERS.

A considerable sensation has been produced by a recent trial, before Lord Tenterden, in which Mr. Handey recovered a bill of five guineas, charged for a course of medicines and attendance, on the family of Mr. Henson an attorney. The medicines were all specified, and also the visits; but, not the individual or specific charges—merely the sum total. The defendant resisted on the grounds, first that the charges were too high—and secondly that Mr. Handey had no right to charge for visits. Lord Tenterden held that "a general practitioner, who did not send useless quantities of medicine, was entitled to remuneration for his attendance." The jury therefore found for the plaintiff.

This is, no doubt, an important decision, as far as a single precedent goes, and where there is evidently no fixed law on the subject. But it does not set the matter at rest, as some of our more sanguine cotemporaries anticipate. Such a decision as the above would always have been given, and will always be given, by a court of justice, when the demand evinced such moderation and fairness as Mr. Handey's bill bore on its face. But is it to be concluded that, because a jury gave compensation for attendance as well as medicines, the general practitioner has now nothing to do, but pursue the course which Mr. Handey followed? Such a conclusion is very beautiful in the closet; but, as yet, very impracticable. There is another party to be consulted—the patients themselves. Excepting upon very urgent occasions, and one in which a precedent was sought for, nothing can be more dangerous for a medical man than going to law for his remuneration. It must be recollected too, that a patient, although he may not choose to resist the demand of a general practitioner, has the alternative of applying next time, to another surgeon, who acts on a different plan. The fact is, that nothing but a unanimous resolution among practitioners themselves, to adopt the plan of charging for medicine and attendance, can be at all effectual. It is extremely difficult to bring medical men to unanimity in this common cause. Several general practitioners in the immediate neighbourhood of London, have, for some years past, acted on a still better plan than that of Mr. Handey—namely that of making the charge in a single line—" medicine and attendance, from such a date to such a date, such a sum." Still, even here, a few of the older practitioners in the same neighbourhood, hold out for the old plan, and consequently none but those who are firmly and independently established dare to risk the new procedure. We say then, that the law need not, and should not be resorted to, in this case. The general practitioners of each town should meet together, and enter into a resolution to charge for visits, and send a proportionately smaller quantity of medicines. Unfortunately, the jealousies and distractions of medical society offer a strong bar to this community of interest and unity of action, at the present time—and till such simultaneous movement takes place, the judicial decision in Mr. Handey's case will have little or no beneficial effect. Perhaps indeed it may stimulate them to the plan which we have long ago urged on their notice.

